# Glycosphingolipids as emerging attack points in bladder cancer

**DOI:** 10.1007/s12672-025-02302-y

**Published:** 2025-04-19

**Authors:** Inês B. Moreira, Falk F. R. Buettner

**Affiliations:** 1 Institute of Clinical Biochemistry, Hannover Medical School, 30625 Hannover, Germany; 2 Proteomics, Institute of Theoretical Medicine, Faculty of Medicine, University of Augsburg, Universitätsstrasse 2, 86159 Augsburg, Germany

**Keywords:** Bladder cancer, Glycosphingolipids, Biomarkers

## Abstract

Bladder cancer is a prevalent malignancy associated with significant morbidity and mortality worldwide. Emerging research highlights the critical role of glycosphingolipids (GSLs) in bladder cancer progression. In this review, we examine GSL expression profiles in bladder cancer and explore their contributions to key cancer hallmarks, including invasion and metastasis, immune evasion, and resistance to cell death. We further discuss the potential of GSLs as therapeutic targets and non-invasive biomarkers, with an emphasis on recent advances in GSL-targeting strategies. Additionally, we highlight our recent discovery of a novel, patented biomarker for bladder cancer diagnosis, identified using cutting-edge glyco-analytical technologies.

## Introduction

Bladder cancer is the most common malignancy of the urinary tract, affecting nearly 20 million individuals and causing approximately 9.7 million deaths worldwide in 2022 [1]. It is a heterogeneous disease classified into two major subtypes: non-muscle invasive bladder cancer (NMIBC) and muscle-invasive bladder cancer (MIBC). NMIBC accounts for approximately 75% of newly diagnosed cases and is generally associated with a more favorable prognosis [2, 3]. However, disease recurrence is frequent, with up to 50–70% of NMIBC patients experiencing tumor relapse, and 10–30% progressing into MIBC [4, 5]. In contrast, MIBC is more aggressive, with a higher risk of metastasis and significantly worse survival outcomes, contributing to the majority of bladder cancer-related deaths [6].

The current standard of care for bladder cancer includes early diagnosis, surgical resection, and cisplatin-based chemotherapy as primary treatment strategies. However, due to the heterogeneous nature of the disease, these treatment modalities often fail to prevent tumor recurrence and disease progression [7]. Among the available treatment options, Bacillus Calmette-Guérin (BCG) immunotherapy remains the most effective adjuvant treatment for high-risk NMIBC, significantly reducing recurrence and progression rates. However, despite its success, BCG therapy fails in approximately 40% of patients, with many experiencing recurrence or progression after initial response [8]. This highlights the urgent need for novel therapeutic strategies to improve outcomes for BCG-unresponsive patients.

Over the last decades, extensive research efforts have focused on identifying novel biomarkers for early and non-invasive diagnosis, patient stratification, prognosis and targeted therapeutics [9]. One of the most promising biomarker families is glycosphingolipids (GSLs), a class of bioactive glycosylated lipids that play a pivotal role in cellular communication, adhesion and signal transduction [10]. The aberrant expression of GSLs on the cell surface is strongly associated with malignant transformation, impacting tumor cell proliferation, migration, and invasion. Furthermore, GSLs can be actively secreted into bodily fluids, making them promising candidates for non-invasive bladder cancer detection [11].

The complexity of GSL biosynthesis and the challenges associated with their detection have historically limited their application as clinical biomarkers. Nonetheless, recent advancements in glyco-analytical detection methods have renewed interest in GSLs, allowing for more precise and detailed profiling of their expression patterns in bladder cancer. Building on these advancements, this review provides a comprehensive overview of GSL alterations in bladder cancer, examining how their expression patterns differ between NMIBC and MIBC and how these differences influence key aspects of bladder cancer progression, including invasion, metastasis, immune evasion, and therapy resistance. We highlight the biological functions of GSLs, their potential as diagnostic and prognostic biomarkers, and their implications for tumor classification, grading, and therapeutic targeting.

## Composition and function of glycosphingolipids

**Fig. 1:**
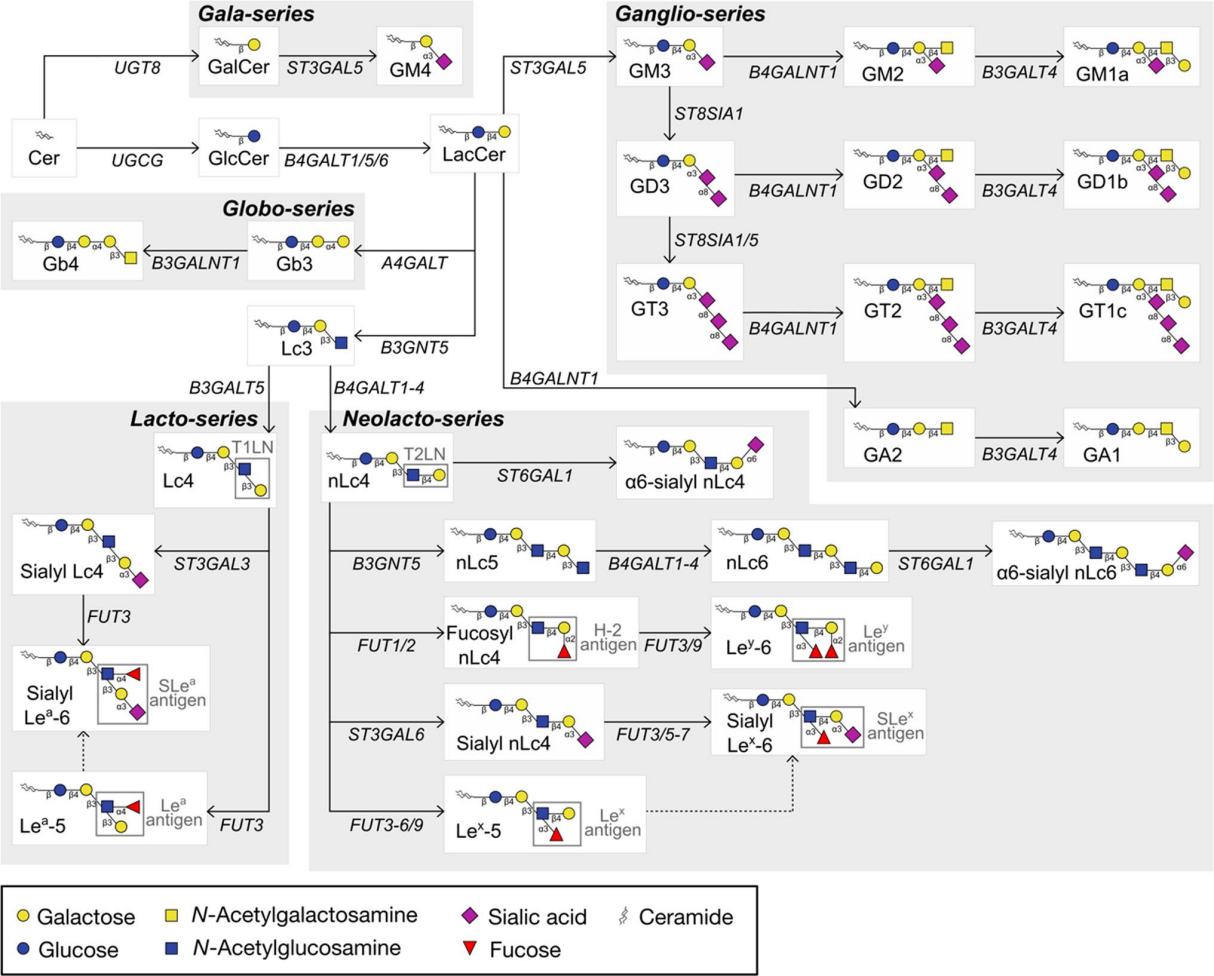
Glycosphingolipid simplified biosynthetic pathways. GSL biosynthesis begins with the glycosylation of a ceramide backbone with either a glucose (to form GlcCer) or a galactose (to form GalCer, precursor of the gala-series). GlcCer is subsequently galactosylated into LacCer, which can be elongated with a sialic acid (to form GM3, precursor of the ganglio-series), a galactose (to form Gb3, precursor of the globo-series) or an *N*-acetylglucosamine (to form Lc3). Lc3 is further galactosylated to form Lc4 (precursor of the lacto-series, β-1,3 linkage) or nLc4 (precursor of the neolacto-series, β-1,4 linkage). The description of the complete pathways is detailed elsewhere [18, 19]. H-2, H type II antigen; T1LN, type I *N*-acetyllactosamine; T2LN, type II *N*-acetyllactosamine

GSLs are a diverse class of glycolipids found on the surface of human cells. To date, hundreds of unique GSLs have been described, each exhibiting distinct glycan/lipid variations. These structures are primarily categorized into specific “series” based on shared glycan core sequences, with the ganglio-, globo-, lacto-, and neolacto-series being the most prominent in vertebrates (Fig. 1) [10].

The biosynthesis of GSLs begins in the endoplasmic reticulum (ER), where a ceramide backbone is synthesized. This lipid molecule is then transported to the Golgi apparatus, where glucosylceramide synthase (GCS) adds a glucose residue to form glucosylceramide (GlcCer), marking the first committed step in the major pathway of GSL synthesis. Lactosylceramide (LacCer) is subsequently generated by the addition of a galactose molecule, and from this point forward, elongation of the GSL structure continues via the secretory pathway through the stepwise addition of glycan moieties by Golgi-resident glycosyltransferases [10].

Once fully synthesized, GSLs are predominantly localized on the outer layer of the plasma membrane, facing the extracellular *milieu* [12]. At the membrane, GSLs undergo continuous turnover through endocytosis and subsequent degradation in lysosomes, where glycosylhydrolases sequentially remove sugar residues from the non-reducing end. A portion of the released monosaccharides and ceramide components is then recycled for de novo glycoconjugate synthesis [10].

In both physiological and pathological conditions, GSLs play a crucial role in cell adhesion, which occurs through interaction with protein binding partners, like galectins, selectins, or other carbohydrate structures. In addition to their action as *trans*-recognition molecules, GSLs also participate in side-by-side (*cis*) interactions, forming clusters composed of either single or multiple GSL species. Rather than being randomly distributed throughout the plasma membrane, these clusters organize into specialized microdomains known as “glycosynapses”. Within these domains, GSLs interact with specific proteins, including growth factor receptors (e.g., epidermal growth factor receptor, EGFR), integrins (e.g., α3β1), tetraspanins (e.g., CD9, CD81) [13–16]. Additionally, they associate with nonreceptor cytoplasmic kinases (e.g., Src family kinases), which are critical regulators of intracellular signaling cascades. These interactions play a crucial role in maintaining cellular communication, modulating membrane dynamics, and coordinating signal transduction in physiological conditions [17].

## Hallmarks of glycosphingolipids in bladder cancer

The search for tumor-associated GSL antigens has been a major focus of investigation for decades. Under normal physiological conditions, GlcCer, LacCer, Gb3 (globotriaosylceramide), Gb4 (globotetraosylceramide), GM3 (monosialodihexosylganglioside) and GD3 (disialodihexosylganglioside) have been identified as the major GSLs present in human bladder tissues [20]. However, during malignant transformation, the cellular membrane composition and organization undergo significant remodeling. As a result, cancer cells exhibit altered GSL expression, which in turn modulates transmembrane signaling pathways essential for tumor growth, invasion and metastasis [21]. These alterations in membrane composition/organization distinguish cancer cells from normal ones. Consequently, targeting such changes has been explored as a potential strategy for improving bladder cancer diagnosis and treatment.

Aberrant glycosylation, including altered GSL biosynthesis, is one of the hallmarks of malignant transformation and cancer progression [22]. Several key alterations in GSL expression patterns have been identified and extensively studied in bladder cancer (Table 1). Notably, GSL expression profiles differ between NMIBC and MIBC, suggesting their potential utility in subclassification and disease monitoring. NMIBC is predominantly characterized by a significant accumulation of GM3, whereas high-grade MIBC exhibits a shift toward higher levels of LacCer, Gb3, Gb4, and GD2 (disialotrihexosylganglioside) [23–25]. These differences indicate that specific GSL signatures are associated with disease progression, making them valuable markers for stratifying bladder cancer subtypes. Understanding these glycosylation patterns not only aids in bladder cancer classification and grading, but may also contribute to refining risk assessment and identifying new therapeutic targets.

**Table 1:** Roles of GSLs and GSL-related molecules in bladder cancer

Glycan component	Sample type/Tumor category	Modification	Relevance/impact	Refs.
GlcCer	Cell lines^b^	Brefeldin A treatment enhances GlcCer expression and decreases invasion	Potential therapeutic agent	[43]
LacCer	Tissue: NMIBC, MIBC, normal; Cell lines^a,b^	Increased in MIBC bladder cancer compared to NMIBC/normal urothelium	Invasion and metastasis	[23] [44]
	Cell lines^b^	Brefeldin A treatment enhances LacCer expression and decreases invasion	Potential therapeutic agent	[43]
*Ganglio-series*				
GM3	Cell lines^a,b^; Mouse model	Antitumor effect: decreased proliferation/motility, cell adhesion/invasion, tumor growth, increased apoptosis	Potential therapeutic agent	[16]
	Cell lines^a,b^	High GM3 level decreases cell motility/invasiveness; Low GM3 level enhances cell motility/invasiveness; Glycosynapse reorganization	Potential therapeutic agent	[45]
	Tissue: NMIBC, MIBC, normal; Cell lines^a,b,c^	Upregulation of GM3 synthase and massive GM3 accumulation in NMIBC bladder cancer	Potential therapeutic agent	[29] [23]
	Cell lines^b^	Brefeldin A treatment enhances GM3 expression and decreases invasion	Potential therapeutic agent	[43]
GM2	Cell lines^c^	Inhibits EtDO-P4/TGF-β induced EMT alterations	Potential inhibition of TGF-β-dependent cell motility	[46]
	Cell lines^a,b,c^	Expressed in bladder cancer cells; GM2/CD82 complex inhibits cell motility through blocking of HGF-induced cMet activation	Potential inhibition of HGF-dependent cell motility	[15] [45] [14]
GD2	Tissue: low/high grade tumors Cell lines^a,b^	Increased in MIBC cells and high grade bladder cancer; GD2 positive cells display cancer stem cell properties and an EMT-positive phenotype	Invasion and metastasis	[24, 25]
	Cell lines^a,b,c^	Anti-GD2 antibody combined with Niraparib increases inhibition of bladder cancer cells	Potential therapeutic approach	[47]
GM2/GM3 heterodimer	Cell lines^b,c^	GM2/GM3/CD82 complex strongly inhibits cell motility through blocking of HGF-induced cMet activation	Potential inhibition of HGF-dependent cell motility	[15]
GA1 (Gg4)	Cell lines^c^	Inhibits TGF-β induced EMT alterations	Potential inhibition of TGF-β-dependent cell motility	[46]
*Globo-series*				
Gb3	Tissue: NMIBC, MIBC, normal;	Increased in MIBC; Upregulation of Gb3 synthase in MIBC	Invasion and metastasis	[23]
	Tissue: tumors; Cell line^b^	Verotoxin 1 binds to Gb3 in endothelial and tumor cells	Cellular target for Verotoxin 1 anticancer drug	[48]
Gb4	Tissue: NMIBC, MIBC, normal;	Increased in MIBC	Invasion and metastasis	[23]
*Lacto-series*				
Le^a^	Tissue: tumors	Distinctive expression patterns at an early neoplastic stage	Diagnosis and prognosis	[33]
Sialyl Le^a^ (CA19-9)	Serum: NMIBC/ MIBC patients, controls;	Increased in invasive and metastatic bladder cancer	Prognosis	[40]
	Urine: NMIBC/ MIBC patients, controls;	Increased in bladder cancer	Diagnosis	[39]
	Cell lines^a,b^	Involved in E-selectin-mediated adhesion	Invasion and metastasis	[49]
	Tissue: tumors	Positive expression in 76% of tumors	Diagnosis	[38]
*Neolacto-series*				
Le^y^	Tissue: CIS/non-CIS patients	Distinctive expression patterns in CIS and non-CIS conditions	Diagnosis	[34]
Le^x^	Urine: patients and controls	Immunocytology shows higher sensitivity than BTA stat and NMP22 tests	Surveillance of high-risk population	[50]
	Urine: patients and controls	Expressed in transitional cell tumors regardless of the grade, stage or secretor status; Absent from normal urothelial cells	Diagnosis	[35]
Sialyl Le^x^	Tissue: NMIBC, MIBC; Cell lines^a,b,c^	Increased in MIBC; Correlated with E-selectin dependent adhesion	Invasion and metastasis	[29] [43] [49]
	Tissue: NMIBC, MIBC; Cell lines^a,b,c^	Increased in MIBC; Higher expression correlated with metastatic phenotype, poor prognosis; Upregulation of *FUT6* and *FUT7* on invasive cells	Invasion and metastasis	[29]
nLc4	Tissue: NMIBC, MIBC, normal; Urine: patients and controls	Increased in bladder cancer tissue and urine	Diagnosis; Potential therapeutic target	[28] [51]
α2,6-sialyl nLc4/6	Cell line^a^	Expressed in NMIBC; Functional receptors for rViscumin, involved in B-chain-dependent signal triggering	Cellular targets for rViscumin Anticancer drug	[52]
*Glycosylation enzymes*				
GCS	Tissue: NMIBC, MIBC, normal	Increased in bladder cancer; higher expression correlates with high grade, metastatic phenotype, poor prognosis	Development and prognosis	[26] [27]
Ganglio-series genes	TCGA databases	Involved in the regulation of ITGB4 splicing events by JUP	Potential role in bone metastasis	[53]
*B4GALNT1*	Public databases; Tissue: MIBC	High expression correlates with clinical stages, poor prognosis, extracellular matrix remodeling and activation of extracellular exosomes in invasive bladder cancer	Accelerates tumor progression	[32]
*ST3GAL5*	Public databases; Cell lines^a,b,c^; mouse model	Downregulated in bladder cancer cells and tissues vs. normal-adjacent tissues; Overexpression inhibited bladder cancer occurrence and progression	Prognosis; Potential therapeutic target	[41]
*ST3GAL6*	Public databases; Cell lines^a,b^	Increased gene expression correlated with tumor stage, grade and survival; Regulated negatively by GATA3 and involved in cell migration and invasion	Biomarker for prediction of poor outcomes; Invasion and metastasis	[54]
*ST8SIA1*	Public databases; Cell lines^a,b,c^	Downregulated in bladder cancer cells and tissues vs. normal; Expression negatively associated with pathological grade and invasiveness of bladder cancer; Antitumor effect mediated by the JAK/STAT signaling pathway	Diagnosis; Potential therapeutic target	[42]
*FUT4*	Cell lines^a,b,c^	Increased gene expression; Increased cell proliferation, migration and invasion, inhibited apoptosis	Tumor promotor; Potential therapeutic target	[36]
*FUT7*	Public Databases; Serum: patients/ controls Tissue: patients/ controls; Cell lines^a,b^	High expression correlates with poor prognosis; Increased in bladder cancer tissue/serum samples; Upregulation promotes cell proliferation, invasion and EMT	Diagnosis and prognosis; Tumor promotor; Potential therapeutic target	[37]
NEU3	Tissue: tumor/normal Cell lines^a,b,c^	Increased gene expression; Promotes the invasiveness of bladder cancer activating ERK and PI3K signaling	Potential therapeutic target; Invasion and metastasis	[55]
*Glycan-binding proteins*				
Galectin-1	Tissue: low/high grade tumors, normal	Increased expression levels in high-grade bladder cancer compared to low grade/normal urothelium	Immunosuppressor agent; Potential use of inhibitors as anti-metastatic agents	[56]
	Cell lines^a,b^	Increased in invasive bladder cancer	Diagnosis	[57]
Galectin-3	Serum: low/high grade tumors, normal	Increased in bladder cancer	Diagnosis	[58]
	Cell line^b^	Inhibits TRAIL-induced apoptosis through elevation of Akt activity	Protection from apoptosis	[59]
	Tissue: low/high grade tumors, normal	Increased expression levels in bladder cancer compared to normal urothelium	Immunosuppressor agent; Potential use of inhibitors as anti-metastatic agents	[56]
Galectin-7	Tissue: MIBC, normal; Cell lines^a,b^	increased expression in chemosensitive patients compared to chemoresistant ones	Overcome chemoresistance	[60]
Galectin-8	Tissue: NMIBC, MIBC, normal	Decreased in MIBC compared to NMIBC/normal urothelium	Tumor inhibitor; Potential therapeutic agent	[61]
*Inhibitors*				
EtDO-P4	Cell lines^c^	Enhances haptotactic cell motility; Conversion from epithelial to fibroblastic cell morphology; Induces EMT	Invasion and metastasis	[46]
	Cell lines^a,b^	Conversion of NMIBC cancer cells into MIBC; Increased Src kinase activity	Invasion and metastasis	[45]

^a^Cell lines established from a patient with NMIBC

^b^Cell lines established form a patient with MIBC

^c^Cell lines established from normal urothelium of a healthy individual

The glycosyltransferase GCS, encoded by the *UGCG* (UDP-glucose ceramide glycosyltransferase) gene, is responsible for the initial glycosylation step leading to the synthesis of all glucose-derived GSLs. Overexpression of GCS has been observed in bladder cancer tissues, where it correlates with poor prognosis [26, 27]. In a recent study, we found that nLc4 (neolactotetraosylceramide), Gb3 and the GM3 ganglioside are significantly increased in bladder tumor tissues when compared to the normal adjacent mucosa and to normal bladder tissues from cancer-free individuals [28]. In different studies, and looking specifically into superficial bladder tumors, these exhibit a significant accumulation of GM3 [29, 30], accompanied by the upregulated expression of GM3 synthase and downregulation of Gb3 and GD3 synthases activity [23].

In the Cancer Genome Atlas Urothelial Carcinoma (TCGA-BLCA) cohort [31], high levels of *B4GALNT1 (*beta-1,4-*N*-acetyl-galactosaminyltransferase 1) expression have been strongly linked to advanced clinical stages and poor prognosis [32]. This gene encodes the glycosyltransferase responsible for the synthesis of ganglio-series GSLs, including GM2 (monosialotrihexosylganglioside), GD2, and GT2 (trisialotrihexosylganglioside), as well as GA2 (asialo-GM2, Gg3). Moreover, the genes *B4GALT3* and *B4GALT4* (beta-1,4-galactosyltransferases 3 and 4), involved in the biosynthesis of nLc4, have been found to be upregulated in bladder cancer compared to normal adjacent tissues [28].

The expression of Lewis antigens has been extensively studied in bladder cancer. Among type-1 fucosylated glycans, Lewis A (Le^a^) expression undergoes changes at early neoplastic stages, and its detection allows for the subclassification of histologically similar tumors into distinct prognostic groups. These characteristics make Le^a^ a valuable biomarker for the diagnosis of very early premalignant urothelial alterations and assessing malignant potential in bladder cancer [33].

Among type-2 fucosylated glycans, Lewis Y (Le^y^) exhibits distinct expression patterns in carcinoma in situ (CIS) compared to non-CIS conditions, making it a useful marker for histological differentiation [34]. Lewis X (Le^x^), which is typically absent from normal urothelial cells in adults, is present in urothelial tumors regardless of tumor grade, stage, or secretor status [35].

Additionally, the expression of fucosyltransferases (FUT) involved in the synthesis of Lewis antigens (FUT1-7 and FUT9) can also be altered in bladder cancer. *FUT4* is overexpressed in multiple bladder cancer cell lines, promoting neoplastic cell proliferation and invasion [36]. *FUT6* and *FUT7* show increased expression in invasive cells compared to noninvasive ones [29]. A recent study further confirmed that *FUT7* expression is elevated in bladder cancer tissues relative to normal tissues and is associated with poor prognosis. This upregulation was validated through immunohistochemical analysis and ELISA-based detection of FUT7 in the serum of bladder cancer patients, reinforcing its potential as a biomarker for bladder cancer detection [37].

The sialylation status of glycans in bladder cancer is directly linked to tumor progression. Sialyltransferases involved in the biosynthesis of sialylated glycans have an active role in providing clues to understand the mechanisms of tumorigenicity. Sialyl Lewis X (SLe^x^), generated by the enzymatic transfer of sialic acid to the Le^x^ antigen, is highly expressed in invasive urothelial tumors and cell lines but absent in noninvasive cells [29]. Similarly, Sialyl Lewis A (SLe^a^), also known as carbohydrate antigen 19-9 (CA19-9), is detected in bladder carcinoma tissues [38] and is elevated in the urine of bladder cancer patients [39]. Notably, serum levels of SLe^a^ are significantly increased in patients with metastatic or advanced bladder cancer, suggesting its potential utility in monitoring disease progression [40].

The expression of sialyltransferases is also altered in bladder cancer. ST3GAL5 (also called GM3 synthase) is downregulated in tumor tissues compared to adjacent normal bladder tissues, with its expression levels higher in low-grade tumors than in high-grade bladder cancer [41]. Conversely, the mRNA expression levels of *ST8SIA1* (the enzyme that converts GM3 to GD3) are significantly decreased in bladder cancer tumors compared to normal bladder tissues, and its low expression is associated with increased pathological grade and invasiveness [42].

**Fig. 2:**
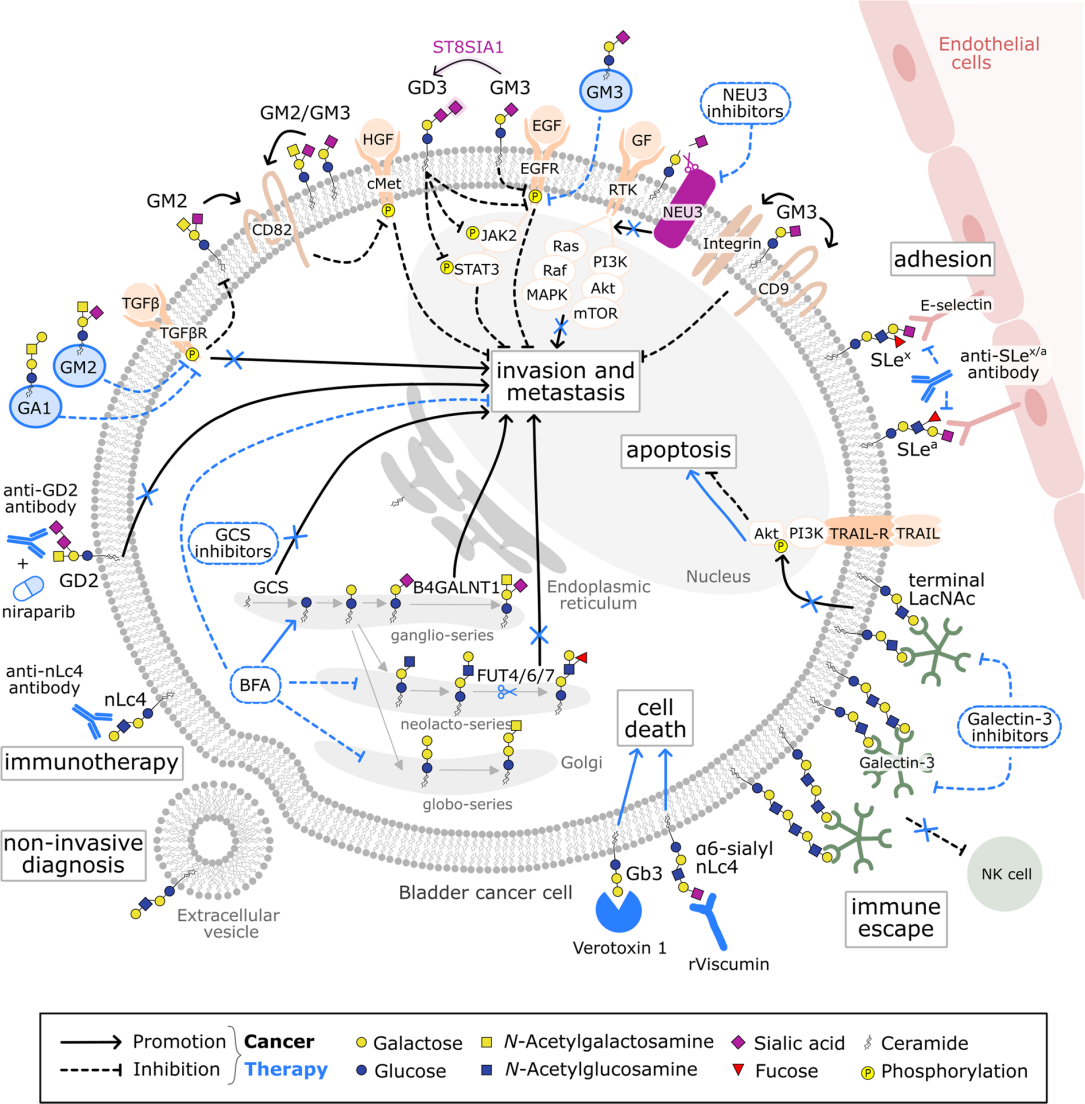
Glycosphingolipid dynamics during bladder cancer progression. The repertoire and content of GSLs can influence different hallmarks of bladder cancer. Regarding invasion and metastasis, overexpression of GD2 is linked to an increased expression of mesenchymal genes, having a pro-metastatic role [24, 25]; exposure of bladder cancer cells to TGF-β decreases GM2 expression and leads to the acquisition of EMT features [46]; overexpression of NEU3 promotes invasiveness of bladder cancer through the activation of ERK and PI3K signaling [55]; high presence of GCS [26] and B4GALNT1 [32] is correlated with a metastatic phenotype; upregulation of *FUT*7 enhances migration and invasion capabilities of bladder cancer cells [37] and SLe^x^ and SLe^a^ antigens mediate E-selectin-dependent adhesion of some bladder tumor cells to microvascular endothelial cells, increasing the metastatic potential [49]. Regarding invasion and metastasis inhibition, GM3 mediates the interaction of α3β1 integrin with CD9 [13] and suppresses the tyrosine phosphorylation levels of EGFR, reducing cell proliferation, motility and adhesion [16]; ST8SIA1 overexpression decreases the phosphorylation of EGFR, JAK and STAT3 by increasing GM3’s sialylation, which inhibits invasiveness [42]; and the interaction of GM2 [14] or GM2-GM3 heterodimer [15] with CD82 inhibits HGF-induced activation of c-Met, impairing cell motility. GSL-related molecules can also have a role in regulating tumor cell apoptosis. Overexpression of galectin-3 promotes the activation of the PI3K/Akt pathway, inhibiting TRAIL-induced apoptosis [59]. The interaction of galectin-3 with poly-LacNAc-containing glycans on the surface of tumor cells can mask them from NK cells, facilitating immune evasion and tumor progression [56, 62]. Overall, these biological processes highlight the contribution of GSLs as promising immunotherapeutic targets or drug agents for bladder cancer treatment. This includes the administration of GSLs and GSL modulators/inhibitors, the use of antibodies or anti-cancer drugs that target GSLs, and the manipulation of genes involved in GSL biosynthesis through genetic engineering

These bladder cancer-specific alterations in the composition and repertoire of GSLs contribute to multiple hallmarks of cancer, including activation of invasion and metastasis, immune evasion, and resistance to cell death. These features will be explored in detail in the next subsections of this review and are summarized in Fig. 2.

### Activating invasion and metastasis

Epithelial-mesenchymal transition (EMT) is a process in which epithelial cells lose polarity, integrity and adhesion, adopting a mesenchymal phenotype that enhances their migratory capacity. The acquisition of EMT by cancer cells is characterized by the loss of E-cadherin and upregulation of mesenchymal markers such as N-cadherin, fibronectin and vimentin. This transition is driven by multiple growth factors, including epidermal growth factor (EGF), hepatocyte growth factor (HGF), and transforming growth factor-β (TGF-β) [63].

Specific GSLs interact with different signal transducers, growth factors and integrins, playing key roles in EMT regulation and tumor cell migration [15, 45, 46]. Recent evidence further supports the role of GSLs in facilitating cancer cell plasticity, including our contribution to a study demonstrating GSL involvement in ovarian cancer EMT dynamics [64].

Bladder carcinoma cells treated with TGF-β undergo EMT, exhibiting fibroblast-like morphology, reduced epithelial marker expression, and increased motility. This transformation is accompanied by a significant reduction in GM2 levels, suggesting that specific GSLs regulate EMT-associated motility [46]. Interestingly, treatment with EtDO-P4 (GCS inhibitor) induced similar EMT effects as TGF-β stimulation, likely due to the depletion of motility-suppressing GSLs. Supporting this hypothesis, exogenous addition of GM2 reversed the motility enhancement caused by EtDO-P4, while GA1 (asialo-GM1, Gg4) inhibited TGF-β-driven motility changes [46].

Furthermore, high-grade bladder cancer tissues overexpressing GD2 also showed increased mesenchymal marker expression, including N-cadherin and vimentin, indicating an EMT-positive phenotype. GD2-positive cells also exhibited cancer stem-like characteristics, correlating with elevated CD44 expression, which reinforce the role of GD2 in tumor progression and metastasis [24, 25, 65].

Single-cell RNA sequencing data from the TCGA-BLCA cohort and immunohistochemical stainings of bladder tumor tissues showed that the above-mentioned glycosyltransferase B4GALNT1 is enriched at both the RNA and protein levels in cancer-associated fibroblasts (CAFs), which simultaneously express high levels of heat shock proteins [32]. Given that CAFs actively contribute to extracellular matrix (ECM) remodeling and tumor progression, and that heat shock proteins facilitate exosome release [66, 67], this suggests that high B4GALNT1 expression is associated with enhanced ECM reorganization and intercellular communication via exosome-mediated signaling. Consequently, B4GALNT1 is proposed to facilitate tumor-stroma interactions, ultimately driving the progression of MIBC [32].

Similarly, the role of glycosyltransferases in bladder cancer progression is further supported by studies on FUT7. Bladder cancer cell lines engineered to overexpress *FUT7* exhibited significantly increased proliferation, migration, and invasion capabilities, accompanied by an induction of EMT [37].

Cell adhesion and motility are primarily regulated by integrins, which mediate interactions with ECM [63]. Integrin functions can be modulated by their interaction with tetraspanins, which in turn can be mediated by association with gangliosides. CD9, a tetraspanin known to suppress cell motility, requires GM3 as a co-factor for its inhibitory function [13]. In detail, in this glycosynaptic microdomain, GM3 enhances and stabilizes the interaction between CD9 and α3β1 integrin, resulting in a low-motility phenotype.

Beyond adhesion, GM3 levels directly impact c-Src activation, a non-receptor tyrosine kinase that regulates multiple oncogenic signaling pathways. High GM3 expression, characteristic of NMIBC, suppresses c-Src activation, whereas reduced GM3 levels in invasive cells result in c-Src hyperactivation, promoting motility and invasiveness [68]. Restoring GM3 levels in invasive bladder cancer cells enhances CD9/α3β1 interactions, suppresses c-Src activity, and reverses the invasive phenotype [45]. Additionally, GM3 modulates the activity of growth factor receptors, particularly EGFR, by reducing EGFR tyrosine phosphorylation, thereby suppressing EGF-induced signaling and inhibiting proliferation and adhesion [16].

Emerging evidence highlights the role of ECM stiffness in bladder cancer progression and recurrence. Increased ECM stiffness has been observed in bladder tumors, particularly in recurrent cases, and has been linked to enhanced tumor cell proliferation, migration, and invasion [69–71]. ECM stiffening activates β1 integrin, triggering downstream focal adhesion kinase (FAK) and CDC42 signaling, which leads to Yes-associated protein (YAP) activation. In stiffer tumor environments, nuclear YAP drives gene expression programs that promote tumor progression and therapy resistance [69, 71]. While the role of ECM stiffness in bladder cancer is becoming clearer, little is known about how GSLs may participate in this process. Given their roles in cell adhesion, receptor clustering, and signal transduction, GSLs may influence ECM remodeling by regulating integrin dynamics and mechanosignaling pathways. Additionally, alterations in GSL composition may affect the organization of glycosynapses, potentially impacting how tumor cells sense and respond to mechanical cues within the tumor microenvironment. Investigating the crosstalk between GSLs and ECM stiffness could provide novel insights into bladder cancer progression and reveal new therapeutic opportunities.

Focusing on sialyltransferases, downregulation of *ST3GAL5* (the gene that codes for the glycosyltransferase that converts LacCer into GM3) has been linked to increased proliferation, migration and invasion of J82 bladder cancer cells, whereas its overexpression suppressed tumor progression of T24 and 5637 cells [41]. In vivo, upregulation of *ST3GAL5* significantly inhibited the tumorigenicity of bladder cancer cells subcutaneously inoculated into BALB/c nude mice [41].

Conversely, increased expression levels of *ST8SIA1* (the gene that codes for the glycosyltransferase that converts GM3 into GD3), was associated with reduced proliferation, migration, and invasion of bladder cancer cells [42]. Mechanistically, *ST8SIA1* overexpression inhibited Janus kinase 2 (JAK2) and signal transducer and activator of transcription (STAT3) phosphorylation, leading to a significant decrease in downstream effectors involved in tumor progression, including matrix metalloproteinase-2 (MMP2), proliferating cell nuclear antigen (PCNA), cyclin D1, and B-cell lymphoma 2 (BCL2) [42]. Because GM3 has been reported to suppress EGFR phosphorylation in bladder cancer cell lines [16], it was suggested that further sialylation of GM3 by ST8SIA1 may enhance this inhibitory effect by reducing EGFR, JAK, and STAT3 phosphorylation levels.

Another important glycosynaptic microdomain involves c-Met (the receptor for HGF), tetraspanin CD82, integrins, and GSLs. c-Met is a critical growth factor receptor that controls cell motility and growth. Studies have shown that GM2, rather than GM3, preferentially interacts with CD82, inhibiting HGF-induced c-Met activation and suppressing bladder cancer cell motility [14]. Further investigations revealed that a combination of GM2 and GM3 within the CD82 complex enhances c-Met suppression, leading to a stronger inhibitory effect on cell motility [15].

NEU3 (Neuraminidase 3) is a plasma membrane-associated ganglioside-specific sialidase that hydrolyzes GM3 into LacCer. *NEU3* is significantly upregulated in bladder cancer tissues compared to normal ones, and its overexpression has been shown to promote tumor cell invasion [55]. Mechanistic studies using siRNA-mediated *NEU3* knockdown revealed that reducing *NEU3* expression suppressed extracellular signal-regulated kinase (ERK) and PI3K signaling, disrupting key pathways involved in tumor progression. These findings suggest that NEU3 functions upstream of Ras/MAPK and PI3K/Akt signaling to promote bladder cancer aggressiveness [55].

An additional study using TCGA and TCGASpliceSeq databases identified genes from the GSL biosynthesis ganglio-series pathway as key regulators of bladder cancer bone metastasis. This glyco-pathway was linked to the co-expression of integrin beta 4 (ITGB4), which is regulated by junction plakoglobin (JUP) [53]. Briefly, ITGB4 phosphorylation in bladder cancer tissues is associated with the activation of EGFR family signaling pathways involved in tumorigenesis and metastasis. In bladder cancer patients with bone metastasis, JUP downregulates ITGB4 splicing events via the ganglio-series biosynthesis pathway, further supporting the role of GSLs in metastatic progression [53].

Binding of selectins to terminal SLe^x^ and SLe^a^ antigens initiates one of the earliest steps in the metastasis cascade [72]. In bladder cancer, E-selectin-dependent cell adhesion was significantly inhibited in vitro by anti-SLe^x^ and anti-SLe^a^ antibodies [43, 49]. Additionally, SLe^x^-positive bladder cancer patients exhibited higher rates of regional lymph node and distant metastasis than SLe^x^-negative patients, likely due to enhanced E-selectin-mediated adhesion of tumor cells to microvascular endothelial cells [29]. This underscores the critical role of SLe^x^ in promoting the invasive and metastatic potential of bladder cancer.

ST3GAL6, the enzyme that has α-2,3-sialyltransferase activity toward the Gal-β1,4-GlcNAc structure on glycoproteins and glycolipids, has high specificity for nLc4 and nLc6, contributing to the formation of a terminal SLe^x^ epitope. Analysis of TCGA-BLCA gene expression data revealed that elevated *ST3GAL6* levels were positively correlated with advanced tumor stage, higher tumor grade, and reduced overall survival in bladder cancer patients [54]. Furthermore, *ST3GAL6* overexpression enhanced bladder cancer cell migration and invasiveness, whereas *ST3GAL6* knockdown suppressed these aggressive features. Interestingly, *ST3GAL6* expression appears to be negatively regulated by GATA3 (GATA-binding protein 3), a transcription factor crucial for maintaining luminal differentiation in urothelial cells. Loss of GATA3 in non-luminal bladder cancer subtypes may relieve this repression, leading to increased *ST3GAL6* expression and a more invasive tumor phenotype [54].

### Evading immune destruction

Another hallmark of bladder cancer progression is the formation of an immunosuppressive and tolerogenic tumor microenvironment, which allows malignant bladder epithelial cells to evade immune surveillance [73]. Tumors can escape immune recognition through multiple mechanisms, including the active secretion of specific GSLs molecules, which disrupt normal immune function [21].

The secretion of GSLs from tumor cells is significantly higher than from normal cells, occurring in the form of membrane vesicles, micelles or monomers [21, 74]. Once released, tumor-derived GSLs can bind to host immune cells in the tumor microenvironment, suppressing key antitumor immune responses, including antigen processing and presentation, lymphocyte proliferation, and cytotoxic effector function. This creates an immune-suppressive niche, promoting tumor growth and immune evasion [21].

A study analyzing gene-expression data from bladder cancer patients revealed that *ST3GAL5* expression significantly modulates the tumor immune microenvironment. Notably, *ST3GAL5* expression was inversely correlated with M0 macrophage infiltration and positively correlated with regulatory T-cell (Treg) infiltration. Low-*ST3GAL5* expressing tumors exhibited a higher abundance of immune-infiltrating cells, which may contribute to improved patient prognoses [41].

Galectins are carbohydrate-binding proteins that recognize galactose-β1,4-*N*-acetylglucosamine (type II LacNAc) motifs found on different surface glycoconjugates, including GSLs of the neolacto-series (e.g., nLc4) [75]. In the context of cancer, galectins play key roles in angiogenesis and immune modulation, facilitating tumor immune escape [76]. Among these, galectin-3 has emerged as a promising diagnostic and prognostic marker in bladder cancer. Serum levels of galectin-3 were found to be significantly elevated in bladder cancer patients compared to controls, with strong correlations to tumor type, stage and grade [58]. Similarly, bladder tumor tissues exhibit higher galectin-3 expression compared to normal urothelium, further supporting its role in tumor progression [56].

Functionally, galectin-3 binds to multiple sites along poly-LacNAc chains on the tumor cell surface, effectively shielding tumor cells from immune recognition and destruction by natural killer (NK) cells. This interaction highlights a crucial mechanism by which bladder cancer cells evade immune surveillance and establish an immune-suppressive microenvironment [62].

Similarly, galectin-1 expression is markedly increased in high-grade bladder tumors compared to low-grade tumors or normal bladder tissues, underscoring its role as an immunosuppressive factor and tumor promotor [56]. Using a multiplexed immunosensor, galectin-1 was found to be increased in invasive bladder cancer cell lysates, suggesting its potential as a biomarker for distinguishing different bladder cancer grades [57].

Interestingly, in contrast to galectin-3 and galectin-1, galectin-8 appears to play an opposing role in bladder cancer progression. Detection of galectin-8 was significantly lower in invasive bladder tumors compared to superficial bladder cancer or normal urothelium, suggesting that galectin-8 loss is associated with more aggressive bladder cancer phenotypes [61]. This implies that galectin-8 may negatively regulate tumor progression by modulating cell adhesion and growth. However, further research is needed to clarify its specific functional role in bladder cancer.

Despite the well-established role of galectins in bladder cancer immune evasion, their direct interactions with specific GSLs in this malignancy remain largely unexplored. Given that GSLs play important roles in shaping the immune microenvironment, future studies should investigate how GSL-galectin interactions influence immune cell recruitment, activation, and suppression in bladder cancer.

### Resisting cell death

An additional function of galectins in cancer is their role in regulating tumor cell survival and apoptosis. Resistance to apoptosis is a critical survival mechanism that enables tumor cells to evade programmed cell death, whether induced by immune effector cells such as NK and cytotoxic T lymphocytes, or by anticancer therapies [77].

In bladder cancer, galectin-3 functions as an anti-apoptotic molecule, promoting tumor cell survival. Overexpression of galectin-3 in invasive bladder cancer cells has been shown to inhibit apoptosis induced by the tumor necrosis factor-related apoptosis-inducing ligand (TRAIL) [59]. Mechanistically, galectin-3 enhances activation of the PI3K/Akt pathway, leading to Akt recruitment to the plasma membrane. Once activated, Akt phosphorylates key downstream targets, effectively blocking apoptotic signaling and promoting cell survival [59].

Galectin-7 has been identified as a potential predictive marker for cisplatin (*cis*-diamminedichloroplatinum, CDDP) chemosensitivity in bladder cancer. Tumor samples from chemosensitive patients exhibited higher galectin-7 expression levels compared to those from chemoresistant patients [60]. In bladder cancer cells, exposure to CDDP induces the expression of the tumor suppressor protein p53, which subsequently upregulates galectin-7 expression, thereby increasing chemosensitivity to CDDP. Interestingly, in p53-mutant bladder cancer cells, exogenous expression of galectin-7 was sufficient to sensitize cells to CDDP by promoting intracellular reactive oxygen species (ROS) generation [60]. These findings suggest that galectin-7 acts downstream of p53 in bladder cancer cells and may serve as a promising target for overcoming cisplatin resistance.

As previously mentioned, GSLs containing LacNAc motifs are putative galectin targets. Given the role of galectins in apoptosis resistance, GSL-galectin interactions may influence drug response and tumor cell survival in bladder cancer. Further research is warranted to explore how specific GSLs modulate apoptotic pathways and contribute to therapy resistance in this malignancy.

## Glycosphingolipid-based non-invasive markers in bladder cancer

At this point in the review, it is evident that a significant number of studies support the potential of GSLs for patient diagnosis and stratification. However, as most studies differ in cohort size and clinicopathological parameters, while also relying on different types of biological samples, definitive conclusions are still lacking to support the introduction of GSLs in clinical practice. Nonetheless, there are several GSL-related markers that hold promise for the non-invasive detection of bladder cancer, whether for the purpose of early diagnosis, prognostic assessment, or disease follow-up.

During the past decades, numerous urinary biomarkers have been evaluated with the goal of diagnosing bladder cancer more accurately, detecting recurrences, and avoiding unnecessary cystoscopy procedures. The currently approved urinary biomarkers by the Food and Drug Administration (FDA) and European Medicines Agency (EMA) include nuclear matrix protein (NMP22), bladder tumor antigen (BTA), UroVysion and ImmunoCyt/uCyt+. Although commercially available, these tests are not commonly used in daily practice, primarily due to the lack of specific guidelines and insufficient clinical benefits [78]. Therefore, urine cytology, which screens for exfoliated tumor cells in the urine, continues to be the standard choice, despite its suboptimal sensitivity and overall efficacy [7]. Since urogenital cancers shed tumor cells and cell fragments into the urine, including GSLs from apoptotic cancer cells or even from active cellular secretion [21, 74], the identification of tumor-derived GSLs presents an opportunity to improve non-invasive detection methods.

The increasing adoption of telemedicine in urological care, accelerated by the COVID-19 pandemic, has demonstrated significant benefits for bladder cancer management. Telemedicine facilitates remote consultations, improves access to early diagnostic evaluations, and enhances patient monitoring, reducing the need for frequent hospital visits, particularly for high-risk individuals undergoing regular surveillance [79, 80]. Beyond improving clinical accessibility, telemedicine offers a platform for integrating biomarker-based diagnostics, including GSL-based urinary biomarkers. Detecting tumor-derived GSLs in urine could enhance non-invasive bladder cancer detection and real-time disease monitoring, improving diagnostic accuracy and complementing current screening methods. Incorporating these approaches into telemedicine could enable remote sample collection and analysis, streamlining diagnosis and follow-up while reducing patient burden. This synergy between digital health and glycobiology holds promise for advancing early detection and personalized care.

A recent study from our group identified nLc4 as a novel non-invasive biomarker for bladder cancer detection, demonstrating that this glycan is significantly increased in urine samples from patients compared to those from cancer-free individuals or patients with other genitourinary malignancies [28]. Furthermore, studies of human bladder lavage specimens using an anti-Le^x^ antibody identified malignancy with an overall sensitivity of 85%, compared to 62% for urine cytology [81]. Notably, by combining both methods, the sensitivity increased to 93%. The presence of Le^x^ antigen in exfoliated cells from urine samples proved to be useful not only for the diagnosis of bladder cancer but also for predicting tumor recurrence and/or progression [50, 81]. While NMP22, a protein that is elevated in the urine of patients with bladder cancer compared to controls, shows a high rate of false positive results, making it unsuitable for screening purposes [82], its combination with anti-Le^x^ immunocytology has demonstrated a high negative predictive value, potentially enhancing its clinical applicability [83].

Like nLc4 and Le^x^, the urinary detection of additional GSLs could hold significant clinical benefits. To date, however, most studies on GSL biomarker candidates have been carried out using bladder cancer tissue samples and cell lines [23, 29, 43]. Therefore, deciphering the “GSL fingerprint” of both primary bladder tumor biopsies and urine samples from bladder cancer patients could revolutionize the field of biomarker discovery.

## Targeting glycosphingolipids in bladder cancer with therapeutic prospects

In the last few decades, significant progress has been made regarding the use of certain GSLs as therapeutic targets or drug agents for bladder cancer treatment and immunotherapy. The administration of exogenous GSLs represents one such strategy. When introduced into the system, GSLs can be incorporated into cellular membranes, modifying antigenicity and altering growth behavior in the recipient cells [84]. For instance, treatment of bladder cancer cell lines with GM3 has been shown to inhibit tumor cell proliferation, adhesion, motility, and invasiveness [23, 45]. Moreover, the intravesical instillation of GM3 in murine orthotopic bladder cancer models significantly reduced tumor growth [16]. These findings support the potential use of GM3 administration as a therapeutic strategy to inhibit or even reverse the invasive phenotype of bladder cancer cells.

A different way of altering the GSL composition of a cell is through genetic engineering, which enables the manipulation of genes involved in glycan biosynthesis and degradation pathways. GlcCer, in contrast to its precursor ceramide, has been associated with increased tumor cell proliferation and resistance to anticancer agents. Conversely, ceramide itself acts as a tumor suppressor, inducing apoptosis in multiple malignancies, including bladder cancer [27, 85]. Thus, targeting *UGCG* expression represents a promising therapeutic strategy for bladder cancer treatment. Recent studies have further highlighted the central role of GCS in cancer progression and therapy resistance. GCS overexpression is linked to enhanced tumor cell survival and drug resistance mechanisms, emphasizing its potential as a therapeutic target in various cancers [86, 87]. Moreover, inhibiting GCS disrupts glycosphingolipid metabolism, impairing critical signaling pathways associated with tumor proliferation and metastasis [88, 89].

Following the same rationale, stable transfection of murine bladder carcinoma cells with cDNA encoding human GM3 synthase (ST3GAL5) resulted in reduced tumor proliferation, motility, and invasion while promoting apoptosis and suppressing xenograft tumor growth [30]. Additionally, a recent study showed that a low expression level of *ST3GAL5* correlates with increased tumorigenesis and progression of bladder cancer, highlighting its potential as a predictive biomarker as well as a therapeutic target [41].

Because a reduced expression of *ST3GAL5* creates an immunosuppressive tumor microenvironment in bladder cancer patients, with key immune checkpoint genes (CTLA-4, LAG3, TIGIT, PD-1, PD-L1, CD80, CD86 and GZMB) being significantly upregulated compared to high-*ST3GAL5* patients, this potentially enhances their susceptibility to immune checkpoint blockade therapy [41]. The results further demonstrate that the low-*ST3GAL5* group of patients displays greater sensitivity to combined anti-PD-1/PD-L1/PD-L2 and anti-CTAL4 treatments relative to the high-ST3GAL5 group [41].

In addition to immunotherapy, *ST3GAL5* expression levels have also been linked to differential chemosensitivity. The low-*ST3GAL5* group showed decreased sensitivity to methotrexate and an enhanced response to other chemotherapy agents, including cisplatin, docetaxel, doxorubicin, gemcitabine, paclitaxel and vinblastine, when compared to the high-ST3GAL5 group. These findings suggest ST3GAL5 could serve as a biomarker to guide personalized chemotherapy regimens in bladder cancer [41].

Regarding other potential sialyltransferase targets, on one hand, ST8SIA1 may suppress the JAK-STAT signaling pathway, thereby attenuating the proliferation and metastasis of bladder cancer [42]. On the other hand, overexpression of *ST3GAL6* has been shown to promote migration and enhance metastatic potential of bladder cancer cells [54]. Similarly, *FUT4* overexpression has been linked to increased cell proliferation, migration, invasion, and resistance to apoptosis, and *FUT7* overexpression has demonstrated comparable tumor-promoting features [36, 37]. Ultimately, strategies involving *ST3GAL5* and *ST8SIA1* overexpression, or inhibition of *ST3GAL6*, *FUT4* and *FUT7*, could offer new therapeutic avenues for bladder cancer.

As described in the previous section, galectin-7 modulates chemosensitivity of bladder cancer cells to cisplatin [60]. This drug, which remains one of the most potent and widely used anticancer agents, is often associated with the development of chemoresistance, which leads to therapeutic failure and limits its application in bladder cancer patients. Currently, only 35% of metastatic bladder cancer patients initially respond to cisplatin-based chemotherapy, and a significant proportion of those who respond eventually develop resistance [90]. Given this challenge, taking advantage of genetic engineering techniques to enhance galectin-7 expression may represent a novel approach to overcoming cisplatin resistance in urothelial cancer, thereby improving treatment efficacy.

An additional approach that can be explored for the treatment of bladder cancer involves pharmacological modulation of GSL biosynthesis. Brefeldin A (BFA), a macrocyclic lactone that disrupts protein trafficking from the ER to the Golgi apparatus, has been reported to also inhibit de novo synthesis of globo- and neolacto-series GSL core chains in human cells [91]. This modulation of GSL synthesis was shown to induce differentiation and apoptosis of human epithelial carcinoma cells [92]. In bladder cancer, BFA treatment led to significant alterations in GlcCer, LacCer, and GM3 expression levels, ultimately decreasing tumor cell motility and invasiveness [43].

Targeting galectins has gained attention in cancer therapy, with galectin inhibitors demonstrating potential as anti-metastatic agents [93]. Similarly, neuraminidase-inhibitors have shown therapeutic promise, as suppression of NEU3 has been found to inhibit the invasion of aggressive bladder cancer cells, positioning NEU3 as a potential target for treating refractory bladder cancer [55].

GSLs may also be used as direct targets for anti-cancer drugs. For instance, sialylated GSLs have been identified as effective targets for rViscumin (also known as recombinant mistletoe lectin-I), which has demonstrated efficacy in Phase I/II clinical trials in patients with solid tumors [94, 95]. In bladder cancer cells, α2,6-sialylated neolacto-series GSLs, including α2,6-sialyl nLc4 and α2,6-sialyl nLc6, have been identified has primary targets for rViscumin, which acts as an immunomodulator and cytotoxic agent [52].

Similarly, Gb3, the cellular receptor for Verotoxin 1 (produced by enterohemorrhagic *Escherichia coli*), plays a key role in mediating the toxin’s cytotoxic effects on tumor cells. Verotoxin 1 has demonstrated both neoplastic and antiangiogenic properties in vivo [48]. Intratumoral injection of Verotoxin 1 in immunocompromised mice significantly suppressed the growth of bladder cancer xenografts and extended overall survival, effects that were associated with reductions in Gb3 and Gb4 levels [48].

Finally, a recent study evaluated the combined use of an anti-GD2 antibody with the PARP (Poly (ADP-ribose) polymerase) inhibitor niraparib, a drug designed to block DNA repair in tumor cells. This combined therapy significantly reduced bladder cancer cell viability, migration, and invasion, supporting its potential as a novel treatment strategy [47].

In essence, specific GSL targets have already been identified for bladder cancer diagnosis and treatment. However, recent technological advances in analytical tools now allow for a more comprehensive approach to GSL biomarker research. In the next chapter, we explore how these innovations are reshaping the field, moving beyond classical single-GSL studies toward a broader understanding of the GSL signature in bladder cancer. This deeper insight has the potential to unveil new therapeutic targets and refine personalized treatment strategies.

## Discovery of glycosphingolipid-based biomarkers by xCGE-LIF

Multiplexed capillary gel electrophoresis coupled to laser-induced fluorescent detection (xCGE-LIF) is a highly sensitive glyco-analytical tool that enables the identification of disease-related biomarkers [96, 97]. Key advantages of this technology include its exceptional separation capacity, which allows for the precise resolution of structural and linkage isomers at picomolar sensitivity. Moreover, xCGE-LIF is a cost-effective method that supports medium- to high-throughput analyses, with automated data interpretation and analysis workflows [98].

Until now, xCGE-LIF has been widely utilized for glycoproteins analysis [99, 100]. However, we have adapted this technology to facilitate the study of GSLs [101]. Since its adaptation, this novel analytical workflow has proven versatile, enabling the characterization of diverse sample types, including cancer biopsies and cell lines, as well as brain and heart tissues [64, 102, 103].

Recognizing both the advantages of xCGE-LIF and the existing gaps in bladder cancer biomarker research, we applied this technology to analyze tissue and urine samples from bladder cancer patients and controls. Beyond profiling global GSL signatures, this approach successfully identified nLc4 as significantly elevated in patient-derived samples. These findings highlight nLc4 as a promising novel urinary biomarker for bladder cancer detection and a potential immunotherapeutic target [28, 51].

## Concluding remarks and future perspectives

In this review, we provided an in-depth analysis of GSLs as key players in bladder cancer progression, highlighting their roles in tumor invasion, metastasis, immune evasion, and therapy resistance. We also explored emerging GSL-based biomarkers for non-invasive bladder cancer detection and discussed their potential as therapeutic targets. Furthermore, we introduced advanced analytical tools, such as xCGE-LIF, which enable high-resolution GSL profiling, providing new opportunities for biomarker discovery and clinical applications.

Despite this progress, many critical questions remain. While multi-omic studies have significantly enhanced patient stratification and biomarker discovery [104, 105], the GSL glycomic landscape of bladder cancer remains largely unexplored. A deeper understanding of GSL alterations in different bladder cancer subtypes, disease stages, and treatment responses is still needed. Addressing these gaps will be crucial for translating GSL-based findings into clinical applications.

Additionally, the integration of GSL-based biomarkers with other emerging approaches, including liquid biopsy strategies and artificial intelligence-driven data analysis, could enhance diagnostic accuracy and prognostic predictions. Recent research has also revealed the critical role of GSLs in shaping the tumor microenvironment, particularly in immune suppression and therapy resistance. Expanding our understanding of GSL-driven immune modulation and drug resistance mechanisms may facilitate the development of novel combination therapies, including immunotherapy and precision medicine strategies. These efforts will be essential for optimizing treatment selection and overcoming resistance to conventional therapies.

The remarkable progress in glycobiology is reshaping our understanding of GSLs in bladder cancer, offering new opportunities for non-invasive diagnostics, patient stratification, and targeted therapies. Future interdisciplinary efforts integrating glycobiology, computational modeling, and personalized medicine will be key to fully unlocking the potential of GSL-based approaches in bladder cancer management.

## Data Availability

No datasets were generated or analysed during the current study.

## Abbreviations

B4GALNT1: Beta-1,4-*N*-acetyl-galactosaminyltransferase 1
B4GALT3: Beta-1,4-galactosyltransferase 3
B4GALT4: Beta-1,4-galactosyltransferase 4
BCG: Bacillus Calmette-Guérin
BCL2: B-cell lymphoma 2
BFA: Brefeldin A
BTA: Bladder tumor antigen
CA19-9: Carbohydrate antigen 19-9
CAF: Cancer-associated fibroblast
CDDP: *Cis*-Diamminedichloroplatinum
CIS: Carcinoma in situ
ECM: Extracellular matrix
EGF: Epidermal growth factor
EGFR: Epidermal growth factor receptor
EMA: European Medicines Agency
EMT: Epithelial-mesenchymal transition
ER: Endoplasmic reticulum
ERK: Extracellular signal-regulated kinase
FAK: Focal adhesion kinase
FDA: Food and Drug Administration
FUT: Fucosyltransferase
GA1: Asialo-GM1
GA2: Asialo-GM2
GATA: GATA-binding protein 3
Gb3: Globotriaosylceramide
Gb4: Globotetraosylceramide
GCS: Glucosylceramide synthase
GD2: Disialotrihexosylganglioside
GD3: Disialodihexosylganglioside
GF: Growth factor
GlcCer: Glucosylceramide
GM1: Monosialotetrahexosylgangliosde
GM2: Monosialotrihexosylganglioside
GM3: Monosialodihexosylganglioside
GSL: Glycosphingolipid
GT2: Trisialotrihexosylganglioside
HGF: Hepatocyte growth factor
ITGB4: Integrin beta 4
JAK: Janus kinase
JUP: Junction plakoglobin
LacCer: Lactosylceramide
LacNAc: *N*-Acetyl-D-lactosamine
Lc3: Lactotriaosylceramide
Lc4: Lactotetraosylceramide
Le: Lewis antigen
MAPK: Mitogen-activated protein kinase
MMP2: Matrix metalloproteinase-2
NEU: Neuraminidase
NK: Natural killer
nLc4: Neolactotetraosylceramide
NMP22: Nuclear matrix protein
PARP: Poly (ADO-ribose) polymerase
PCNA: Proliferating cell nuclear antigen
PI3K: Phosphoinositide 3-kinase
ROS: Reactive oxygen species
RTK: Receptor tyrosine kinase
SLe: Sialyl Lewis antigen
ST3GAL: ST3 beta-galactoside alpha-2,3-sialyltransferase
ST8SIA1: ST8 alpha-*N*-acetyl-neuraminide alpha 2,8-sialyltransferase 1
STAT: Signal transducer and activator of transcription
TCGA-BLCA: The cancer genome atlas urothelial carcinoma
TGF: Transforming growth factor
TRAIL: Tumor necrosis factor-related apoptosis-inducing ligand
Treg: Regulatory T-cell
UGCG: UDP-glucose ceramide glycosyltransferase
xCGE-LIF: Multiplexed capillary gel electrophoresis coupled to laser-induced fluorescent detection
YAP: Yes-associated protein
